# Development and validation of the Chinese version non-nutritive sweetener FFQ with urinary biomarker in children and adolescents

**DOI:** 10.1017/S136898002200088X

**Published:** 2022-08

**Authors:** Ying-Yueh Chu, Yue-Hwa Chen, Rong-Hong Hsieh, Shih-Min Hsia, Hung-Tsung Wu, Yang-Ching Chen

**Affiliations:** 1 Department of Nutrition and Health Sciences, Chinese Culture University, Taipei, Taiwan; 2 School of Nutrition and Health Sciences, College of Nutrition, Taipei Medical University, Taipei, Taiwan; 3 School of Food Safety, College of Nutrition, Taipei Medical University, Taipei, Taiwan; 4 Graduate Institute of Metabolism and Obesity Sciences, Taipei Medical University, Taipei, Taiwan; 5 Department of Internal Medicine, School of Medicine, College of Medicine, National Cheng Kung University, Tainan, Taiwan; 6 Department of Family Medicine, Taipei Medical University Hospital, Taipei Medical University, No. 252, Wu-Hsing St, Xinyi District, Taipei 11031, Taiwan; 7 Department of Family Medicine, School of Medicine, College of Medicine, Taipei Medical University, Taipei, Taiwan

**Keywords:** FFQ, Non-nutritive sweetener, Reproducibility, Urinary biomarker, Validity

## Abstract

**Objective:**

The purpose of the current study was to develop a validated FFQ to evaluate the intake of non-nutritive sweeteners (NNS) in child and adolescent Asian populations.

**Design:**

Intensive and overall market research was performed to create the applicable NNS-FFQ with thirteen food categories and 305 items. Six intense sweeteners, including acesulfame potassium, aspartame, sucralose, glycyrrhizin, steviol glycosides and sorbitol, were investigated. The validity and reproducibility of the NNS-FFQ were evaluated. The validity was further assessed by examining the consistency of reported NNS intake compared with urinary biomarkers using Cohen’s *κ* analysis.

**Settings:**

This work was considered to be relevant in Asian societies.

**Participants:**

One hundred and two children and adolescents recruited from several clinics were invited to participate in the current study.

**Results:**

High content validity indices and high content validity ratio levels were revealed for each sweetener and food category. Reproducibility among subjects was satisfactory. Significant moderate correlations between estimated steviol glycoside/sucralose consumption and sensitive urinary biomarker levels were demonstrated (*κ* values were 0·59 and 0·45 for steviol glycosides and sucralose, respectively), indicating that the NNS-FFQ can be used to assess an individual’s NNS intake. The dietary intense sweetener consumption pattern evaluated in this measurement was similar to those observed in other Asian countries but differed from those observed in Western populations with respect to types and amounts of NNS.

**Conclusions:**

This validated NNS-FFQ can be an applicable and useful tool to evaluate NNS intake in future epidemiological and clinical studies.

## Graphical abstract

**Figure:**
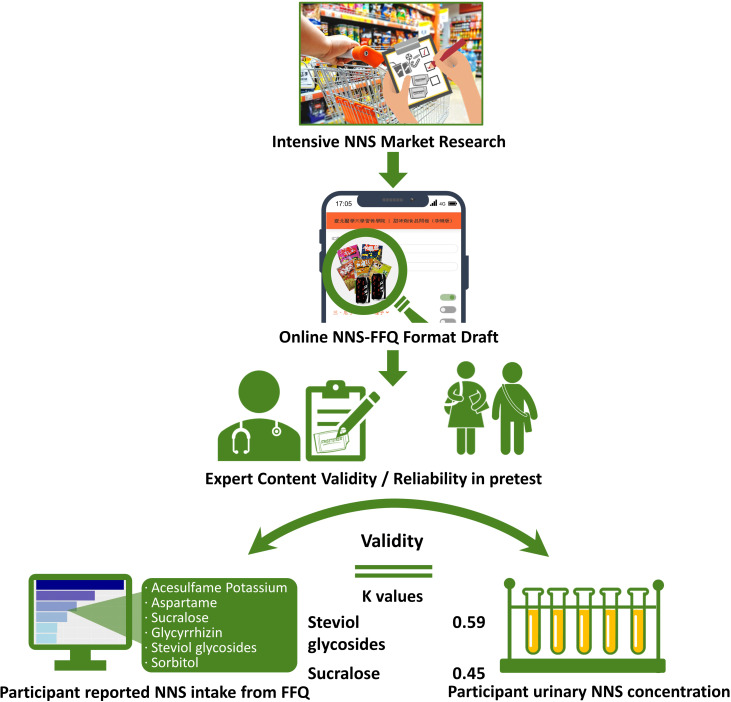

Non-nutritive sweeteners (NNS) are a common food additive used in packaged food to amplify sweetness and increase food palatability^(1)^; they add no or negligible energies. Lower energy intake and weight control were expected when substituting sugar with NNS in food and beverage. However, counterintuitive effects of NNS on metabolic outcomes and comorbidities, such as obesity^(2)^, impaired glucose homeostasis^(3)^, CVD and modification of gut microbiota^(4)^, have been observed.

According to the safety assessment of intense sweeteners by Joint Expert FAO/WHO Committee on Food Additives^(5)^, European Food Safety Authority^(6)^ and US Food and Drug Administration^(7)^, approved NNS were safe for young populations. However, health concerns about NNS intake in children and adolescents were observed in recent studies. No improvement in adverse metabolic conditions was reported after exposure to NNS intake in the early lifespan^(8)^. For instance, BMI in overweight or obese adolescents was not reduced after replacing sugar-sweetened beverages with NNS beverages over a two-year period in a randomised clinical trial^(9)^. Furthermore, positive associations were identified between fat mass increment and NNS drink consumption among children and adolescents in prospective cohort studies^(10,11)^. Early menarche was significantly associated with aspartame-containing drinks or food intake among girls aged 9–10 years in a longitudinal study^(12)^. An increased risk of premature birth^(13)^ and enhanced infant adiposity^(14)^ were also determined to result from constant maternal exposure to NNS during pregnancy. Therefore, the US Institute of Medicine suggests eliminating NNS addition in youth meals and reevaluating NNS use in childhood^(15)^.

Various NNS consumption levels in Asia^(16)^ (e.g. Japan, Korea, China, India and Hong Kong^(17)^) have been measured using national nutritional and health surveys with 24-h dietary recall (DR) or the budget method. The food frequency questionnaire (FFQ) is a typically applied tool for recording the frequency of consumption of foods or nutrients, analysing food patterns and investigating relationships between the nutrients of interest and the health status of individuals and populations over an indicated period and thus may be a more favourable measurement method than DR or a food diary^(18)^. Replying to items listed in the questionnaire is easy and fast for research participants, and the food items generally represent the main sources of nutrients on which the study is focused. Furthermore, a quantitative FFQ can be administered to determine the serving size of participants at each instance of consumption.

Although a validated FFQ was developed to investigate NNS-containing drinks or foods in a US study^(19)^, an FFQ to evaluate NNS intake that is adapted for foods or drinks in Asian populations is lacking. Therefore, in view of the previously developed NNS-FFQ for a US population, constructing and verifying a representing quantitative FFQ to investigate the quality and quantity of NNS consumed by people in Asia are crucial for the following reasons: (1) consumers in Asian populations may engage in diverse dietary behaviours with respect to investigated NNS consumption, whereas beverages are consistently the largest source of NNS intake in Western groups as studies mentioned above^(9–14)^, other food or items were almost omitted. This measure might result in misleading epidemiological conclusions among Asian populations; (2) ingredients and characteristics of packaged food or cuisine in Asian societies’ food products differ considerably from those in Western countries; (3) validating of the FFQ with biomarker is needed and (4) the vulnerable groups of children and adolescents are in the critical developmental periods of life span and possibly more sensitive to stimulus such as food additives compared with adults. The purpose of the study was to establish the Chinese version of quantitative NNS-FFQ for the Asian populations of children and adolescents. The validity of the NNS-FFQ was verified using the urine concentration of the indicated NNS in the study.

## Methods

### Participants

Eligible female children/adolescents aged 6–14 years and male children/adolescents aged 9–17 years were recruited from pubertal and paediatric endocrine outpatient clinics in multiple centres in Taiwan. Compared with healthy adults, children and adolescents were targeted in the current study because biological development in these subjects is susceptible to environmental factors, including foods. Participants were first screened by medical doctors and researchers for eligibility. Subjects with no metabolic disorders and congenital conditions, such as diabetes, hyperlipidemia, maple syrup urine disease and phenylketonuria, were qualified to be included. According to Cade’s report^(20)^, an appropriate sample size for the development and validation of an FFQ should include at least 50–100 research subjects.

### Study design

**Fig. 1:**
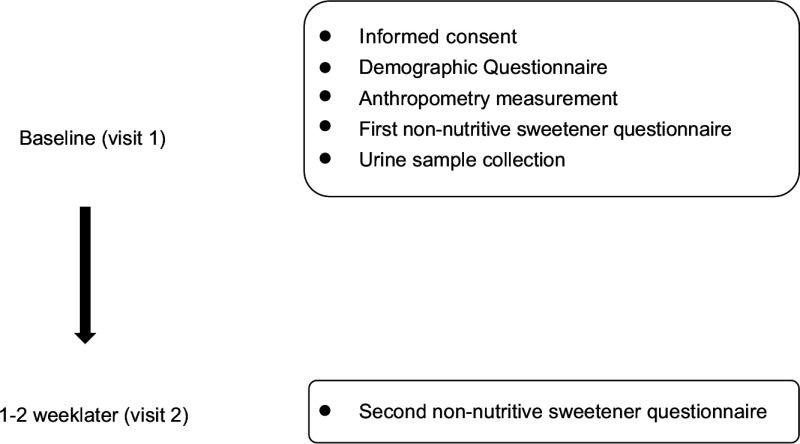
Study design and the timeline for each measurement

Demographic background, FFQ and spot urine data were collected during study recruitment (Fig. 1). Demographic background, including sex, age, race, anthropometric data, education level and household income, was recorded. Body height and weight were measured on a scale of 0·1 cm and 0·1 kg, respectively. BMI was calculated and expressed as kg/m^2^. Cut-off points of age-specific and gender-specific 85th and 95th percentiles were used to define children/adolescents with overweight and obesity, according to the Taiwan Growth Chart^(21)^. The types and amounts of NNS were determined by a database for NNS-containing food reported by manufacturers. Products that did not reveal a clear concentration of NNS were sent to SGS Taiwan Limited—Nankang for analysis of the NNS concentration. The online FFQ was reviewed and completed by participants after three well-trained research assistants explained and assisted aside in case of misreporting due to uncertainty of product recognition and loss of personal patience. Their parents and well-trained assistants were available to aid them in completing FFQ if children and adolescents needed their help.

### Establishment of the quantitative non-nutritive sweeteners-FFQ

Extensive and thorough market research regarding NNS-containing foods at local supermarkets and convenience stores in Taiwan was performed to establish a quantitative FFQ for NNS intake investigation. Beverages, potato or corn chips, cookies, confections, nutritional supplements, frozen foods, dried seasoned seafood and meats, instant noodles, dehydrated and pickled (seasoned) vegetables and fruits and seasoned nuts and seeds containing one or more types of NNS were identified (see online supplemental Table S1). The five major commercial NNS, namely acesulfame potassium, aspartame, sucralose, glycyrrhizin, steviol glycosides and sorbitol, present in food products were screened. The brand name and flavour of each NNS-containing food product were filed, photo-recorded and then classified into thriteen categories with a total of 305 food items on the questionnaire, based on the template created by Myers’ *et al*. The interval scale for frequency was measured as never, ≤1 time/month, 2–3 times/month, 1 time/week, 2–3 times/week, 4–6 times/week, 1 time/d, 2 times/d and ≥3 times/d. The specified serving size was measured at various levels according to online illustrations because actual dietary habits were fairly diverse among people and deviated from manufacturers’ recommended serving suggestions. For example, people usually eat one-half to two packages of instant noodles; however, people chew one to three pieces of gum. To improve recognition of each food product among participants, an online graphic presentation with a food package was designed to reduce the effect of the complexity of brand names and diverse flavours on the presentation of food packages.

### Determination of non-nutritive sweeteners levels in urine

The levels of acesulfame-potassium, sucralose and steviol in spot urine were collected at the first clinic visit. These NNS concentrations were examined using liquid chromatography–MS with an experimental protocol described in a previous study^(22)^. The three indicated NNS standards were provided so that retention time was identified for each analysed component and every test. The excreted concentration of each NNS in urine was first calibrated with creatinine (Cr) level and expressed as ng/mg Cr. The concentration of steviol glucuronide, instead of steviol glycosides, was examined because steviol glucuronide is produced by bacterial hydrolysis in the gut after steviol glycoside intake^(23)^. Therefore, the estimated level of steviol glucosides was determined using a division factor of 0·643, calibrated with the molecular weight of steviol glucuronides and steviol glycosides.

Acesulfame potassium and sucralose, which are not metabolised in the gut, remain intact molecular structures in urine, whereas most aspartame, glycyrrhizin and sorbitol are metabolised completely or partially different into chemicals existing in various tissues. Therefore, aspartame, glycyrrhizin and sorbitol in urine or urinary metabolites cannot reflect the intake level. For instance, most consumed aspartame is hydrolysed into aspartic acid, phenylalanine and methanol. Enoxolone, hydrolysed from glycyrrhizin by bacterial fermentation in the gut, is further degraded in the liver and excreted as bile acids^(24)^. Sorbitol is rapidly converted into dihydroxyacetone phosphate, an intermediate metabolite in the glycolytic pathway^(25)^. By contrast, NNS excreted in urine could be confirmed and quantified over the 24 h following the intake of acesulfame potassium, sucralose and stevia glycosides in preliminary studies performed.

In the preliminary investigation performed in the research group, the concentration of these three NNS excreted in urine increased within 0–5 h after intake and started descending to baseline levels at 24 h after ingestion (see online supplemental Fig. S1). An NNS consumer was defined as one having a urinary NNS concentration greater than a corresponding urinary excretion of ≥ 0·5 % of the acceptable daily intake referring to previous literature^(26)^.

### Statistical analysis

Descriptive statistics were performed to evaluate the demographic data. To verify the face validity of the NNS-FFQ, a panel of ten experts was enlisted to assess the questionnaire content by classifying each food item, frequency interval, serving size and other constructs and variables. The scale content validity index, content validity ratio and *κ* values were used to determine the questionnaire’s applicability. To establish reproducibility in measures, a pretest was performed on both children and adolescents (*n* 91), and Cronbach’s *α* coefficient was calculated. Since Cohen’s *κ* values were quite low between two measures of FFQ1 and FFQ2 among children and adolescents, an independent reproducibility study was conducted by another research group in young adults (aged 18–20 years, *n* 24). The consistency between the two individual questionnaire interviews was examined by Cohen’s *κ* analysis and Spearman correlation, then. The two measures were completed with a 7–14-d interval.

For NNS-FFQ validity demonstrated by urinary biomarkers, both NNS consumption reported from NNS-FFQ and NNS concentration in urine were presented as mean and SD. The percentage was calculated to indicate the proportion of participants exhibiting significant NNS levels in urine and reporting any dietary consumption of specific NNS. Shapiro–Wilk test was performed to analyse normality of FFQ and urinary NNS data and revealed no normal distribution for these data. Cohen’s *κ* analysis was performed to determine the agreement between FFQ responses and urine levels. Statistical significance was set at *P* < 0·05.

## Results

### Demographic background of participants

The child and adolescent age group in the current study had a mean age of 10·61 years; 36 % of 102 participants in this group were boys and 64 % were girls (Table 1); 52 % had a normal BMI, 15 % had an overweight BMI and 12 % had an obese BMI.

**Table 1:** Demographic data for children and adolescents in the study

Characteristics	Children/adolescents (*n* *>)	%
Gender		
Male	37	36
Female	65	64
Age		
Mean	10·61	
sd †	2·17	
BMI (kg/m^-2^)		
Mean	18·00	
sd †	3·06	
Underweight	23	23
Normal weight	52	51
Overweight	15	15
Obese	12	12
Education level	(Parent)	
High school graduate	2	2
College graduate	7	7
University graduate	60	64
Graduate school	25	27
Household income (NTD‡)		
≤30 000	4	4
30 001–59 999	21	23
60 000–99 999	22	24
≥100 000	45	49

* The number of participants does not add up to the total number because of missing data.

† Abbreviation of SD.

‡ Abbreviation of New Taiwan Dollar.

The majority of the children/adolescents’ parents graduated from universities or graduate schools. Household income of over NT$100 000/month was the largest proportion (49 %) in this demographic parameter.

### Non-nutritive sweeteners consumption pattern

NNS consumption measured from NNS-FFQ was shown in both children and adolescents (Table 2). Most of NNS intake came from sucralose, aspartame and acesulfame potassium in these populations. Less than 2 mg intake for each NNS in children and adolescents; therefore, consumption of all five NNS was considerably lower than the upper limit of acceptable daily intake (% acceptable daily intake) set by the Food and Drug Administration of the United States and the Joint Expert FAO/WHO Committee on Food Additives. The proportion relative to acceptable daily intake among the five assessed NNS was small, with ranges of 0·10 %–0·30 % among these subjects.

**Table 2:** Specific and total non-nutritive sweeteners (NNS) consumption reported from the NNS-FFQ for both children and adolescents in the study

NNS type	Amount (mg, mean)	Amount (mg, SD†)	% ADI‡ (mean)	% ADI (SD)
Acesulfame potassium	1·15	5·89	0·18	0·97
Aspartame	1·58	10·11	0·10	0·65
Sucralose	1·74	7·48	0·30	1·26
Glycyrrhizin	0·07	0·25	0·11	0·32
Steviol glycosides	0·25	1·31	0·16	0·79
NNS consumption*	0·96	5·01		
total NNS consumption	4·79	25·04		

* The data represented the mean and SD of the average amount of the five NNS intake listed above.

† Abbreviation of SD.

‡ Abbreviation of average daily intake.

### Face validity of NNS-FFQ

To examine the face validity of NNS-FFQ, the average scale-level content validity indices for both dimensions, each category of food, five types of NNS and sorbitol were ≥ 0·90 (Table 3). Similarly, content validity ratios for both food and sweeteners variables in measures were ≥ 0·80, which indicated agreement among experts. The *κ* values for both foods and sweeteners were considered excellent because all the values were ≥ 0·75, except the value for glycyrrhizin, which was 0·64.

**Table 3:** Validity indices of each intense sweetener and food item by ten experts and reproducibility in children and adolescents (*n* 91) for designed non-nutritive sweeteners (NNS)-FFQ in the pretest

Items of the questionnaire construct	Validity			Reproducibility
	S-CVI* (Average)	CVR† (Average)	*κ* ‡ (Average)	Cronbach’s *α*
Acesulfame potassium	0·99	0·98	0·88	0·44
Aspartame	0·97	0·97	0·83	0·78
Sucralose	0·97	0·96	0·79	0·77
Glycyrrhizin	0·95	0·93	0·64	0·80
Steviol glycosides	1·00	1·00	1·00	0·78
Sorbitol	0·97	0·96	0·78	0·78
Chips, cookies and desserts	0·94	0·93	0·94	0·85
Drinks	0·99	0·98	0·99	0·78
Seaweed	0·90	1·00	1·00	–
Candy and jelly	0·97	0·94	0·97	0·71
Dehydrated minced seafoods	0·97	0·91	0·91	0·95
Frozen foods	0·96	0·97	0·97	0·86
Preserved fruits	0·94	1·00	1·00	0·93
Jerky and dried tofu	0·96	0·96	0·92	0·33
Nutritional supplements	0·90	0·80	1·00	0·76
Seasoned seeds/nuts	1·00	1·00	0·93	0·88
Instant noodles	1·00	1·00	1·00	–

* Abbreviation of scale-level content validity index.

† Abbreviation of content validity ratio.

‡ Abbreviation of Cohen’s *κ* analysis.

### Examination of non-nutritive sweeteners exposure by urinary biomarkers

The excretion levels of acesulfame potassium, sucralose and steviol glycosides in spot urine collection are presented as mean and sd in parentheses (see online Supplemental Table S2). The mean (sd) of acesulfame potassium, sucralose and steviol excreted in the urine for children/adolescents were 9021·38 (27070·82), 444·17 (1180·68) and 1720·08 (4408·64) ng/mg Cr, respectively. These urinary levels explicitly represented daily exposure to NNS in food and drink in the populations. Most likely, detectable NNS concentration in urine reflected what NNS-containing foods or drinks participants had consumed in the 2–24 h prior. Furthermore, large variations among participants were observed for these three detected NNS.

### Validity of the FFQ with urinary biomarkers

Agreement between the reported NNS intake from the FFQ and a significant level of NNS in collected urine was used to distinguish consumers and nonconsumers in Cohen’s *κ* analysis (Table 4). The NNS consumption estimated from the FFQ has exhibited a significant association with urine concentration. The intake of sucralose and steviol glycosides determined from the FFQ measurement was moderately correlated with urine level (0·45 and 0·59 for sucralose and steviol glycosides, respectively). These results indicated that the NNS-FFQ could validly evaluate the assessed NNS consumption in Asian children and adolescents. However, Cohen’s *κ* value for acesulfame potassium was approaching a moderate level (0·40).

**Table 4:** Agreement of a significant non-nutritive sweeteners (NNS) level in urine and NNS intake reported from the NNS-FFQ among children and adolescents using Cohen’s *κ* analysis in the study

NNS type	Percentage of participants with a significant level in urine sample† (%)	Percentage of participants with any consumption reported in FFQ (%)	Cohen’s *κ*
Acesulfame potassium	9	28	0·40*
Sucralose	45	61	0·45*
Steviol glycosides	30	21	0·59*

* *P* < 0·001.

† Significant level is defined as a consumer whose urinary NNS concentration was greater than corresponding urinary excretion ≥ 0·5 % ADI referencing previous literature^(22)^.

### Reproducibility of non-nutritive sweeteners -FFQ

Cronbach’s *α* coefficients in pretest for most food categories (Table 3), NNS and sorbitol, were ≥ 0·70 and were thus determined to be reproducible in children and adolescents, except for jerky (0·33) and acesulfame potassium (0·44). High agreement (≥ 0·61) was demonstrated by Cohen’s *κ* analysis for acesulfame potassium, sucralose, steviol glycosides and sorbitol, but moderate and fair values were observed for aspartame (0·49) and glycyrrhizin (0·35) in the young-adult group, respectively (see online supplemental Table S3). A significant Spearman correlation between two tests indicated good levels for sucralose (0·70) and sorbitol (0·67) and acceptable values (0·36–0·52) for other NNS (see online supplemental Table S4).

## Discussion

### Questionnaire construction

The present NNS-FFQ is the first organised and established FFQ to assess NNS intake among children and adolescents in Asia. Despite NNS exposure being widespread, reported intake from the NNS-FFQ revealed moderate consistency when compared with urinary biomarkers. Content validity was assessed by experts and was determined to be reproducible and valid in both adolescents and children. Reproducibility was moderately consistent. This NNS-FFQ could be a validated tool to assess the levels of exposure to NNS among populations.

NNS-containing processed products are diverse in Taiwan, elevating the complexity and variations of questionnaire responses. In Western societies, soft drinks, milk or milk-related products and confectionery products^(27)^ are known to contain sweeteners as an ingredient in the manufacturing process. Therefore, drinks and some confectionery foods have been considered to be the main samples for inclusion in NNS consumption investigations and related health risk studies^(3–8,10–12)^. In Asia, drinks, candies, cookies and ice cream are not the only major sources of NNS. Preserved foods (tofu, seafood, meats, vegetables and fruits), instant noodles and seasoned nuts and seeds are some of the foods that contain one or more types of NNS. People often consume these products in their daily lives. Both diverse sweeteners and product applications raised the difficulty of the measurement design in the study. To eliminate the problem of product complexity and increase product recognition, food grouping and package photos were included in the questionnaire beside the text on the frequency of consumption and specified portion sizes.

### Characterisation of patterns and amounts of intense sweeteners consumption

Acesulfame potassium was determined to be consumed through four main food categories (thirty four products): soft drinks, frozen foods, seasoned nuts and chewing gums. Numerous food items contained sucralose among the seventy-four products, including potato chips, cookies, confections, drinks, preserved fruits and frozen foods. Notably, six of eight nutritional supplements contained sucralose. Children with malnutrition or other vulnerable populations consume these supplement products to satisfy their daily nutrient needs. Only sixteen products, which were mainly in the potato chips and cookies categories, contained steviol.

Sorbitol consumption was included in this investigation; it is a nutritive sweetener, though. First of all, the intense sweetener with the highest daily intake in adults was determined to be sorbitol in the National Dietary Survey in Japan^(28)^ and South Korea^(29)^ (452 mg/d for Japan and 4·9 mg/kg for South Korea). The main source of intense sweeteners among the investigated sweeteners in this FFQ was also sorbitol. The sorbitol consumption was twenty-three times as totality of NNS for adolescents and children. Sorbitol is mainly used in the production of frozen foods, preserved soybeans, seafood, jerky and nutritional supplements in Japan. In the current study, eight of the thirteen categories included in the measurement contained sorbitol. Close to half of investigated products (150 products): cookies, confections, candies, frozen foods (e.g. dumpling and bun stuffing), preserved jerky, dehydrated tofu and seafood, preserved fruits and nutritional supplements contained intense sweeteners, the most common of which was sorbitol (see online supplemental Table S5).

Second, as countries in East Asia often share common recipes, ingredients and culture in cuisine, packaged foods and daily foods, sorbitol may be widely applied in the processed food industries serving these populations. Furthermore, these products are frequently imported or exported across Asian countries. Over thirty products included in the questionnaire were imported foods from other Asian countries in this investigation. This is another possible reason why sorbitol was the most consumed one among the six examined sweeteners. Notably, only one drink product contained sorbitol. These findings indicate major differences in consumption patterns, including the types of intense sweeteners and foods containing them, between Western and Eastern societies. Populations in the United States and European countries tend to consume high levels of acesulfame potassium, aspartame and sucralose, which were primarily consumed in drinks.

Besides, changes in body composition, metabolic profiles and induced genotoxic effect^(30)^ were observed in the offspring of pregnant and lactating rats treated with sorbitol in the concomitant with a decreased level of fructose and triglyceride in lactating milk. Furthermore, alteration of mammary gland development in 4-week-old mice during the postnatal stage was induced and prolonged after sorbitol administration in pregnant mice mothers^(31)^. Therefore, it is important to explore the consumption pattern and health impact of sorbitol in Asian societies.

### Validity examinations

The validity of each measurement is crucial in demonstrating the applicability of the research. The measurement of biomarkers, such as urinary metabolite level, is necessary to determine the validity. In the current study, Cohen’s *κ* analysis revealed that steviol glycosides and sucralose intake were associated with urinary levels. Chips, cookies and milk tea, the main products containing steviol glycosides, are popular, and their consumption is common among children and adolescents. Sucralose-containing food products (seventy-four items) are widely available in stores. However, acesulfame potassium, which was mostly consumed in soft drinks, was less closely associated with urinary biomarkers, which may be because populations in Taiwan prefer tea or hand-shaken drinks to soft drinks. People may thus consume more steviol glycosides/sucralose-containing products compared with acesulfame potassium-containing products. Therefore, the FFQ of consumers and nonconsumers moderately accorded with the results of the sensitive urinary biomarkers. The validity of aspartame and glycyrrhizin in the questionnaire was not able to be carried out with urinary biomarkers since they would be degraded into metabolites upon intake.

Nondietary factors may influence the relationships between reported intake and urinary biomarkers. Logue *et al.* reported a logarithmic relationship between urine concentration and defined sequential dose treatments of five sweeteners consisting of acesulfame potassium, saccharin, cyclamate, sucralose and steviol in human subjects^(22)^. However, this association was not identified in a recent analysis^(26)^. Instead, a large variation was observed between urinary excretion and intense sweetener intake estimated from 7-d food diaries in a cross-sectional study. Researchers attributed this inconsistency to widespread exposure to NNS from dietary and nondietary sources^(32)^. Furthermore, municipal, wastewater, underground, surface and drinking water sources have been found to be contaminated with NNS and thus may be major sources of NNS^(33)^. Widespread exposure to NNS in foods and the environment may pose an obstacle to the research of intense sweetener intake and related health risks.

### Strengths and limitations in the current study

The applicable NNS-FFQ in the study is the first created for Asian populations. The validity of the FFQ was demonstrated through content validity analysis and confirmed using sensitive urinary biomarkers of steviol glycoside and sucralose. The NNS-FFQ displayed moderate reproducibility. The pattern of intense sweetener intake identified in the NNS-FFQ was similar to that observed in neighbouring countries in studies using national dietary surveys. Therefore, the NNS-FFQ appears to be a useful tool for the evaluation of NNS intake in populations, including vulnerable people such as children and adolescents.

The current study also has several important limitations. First, the study was conducted among vulnerable groups, children and adolescents. It is possible that our findings may not be generalisable to other populations or other countries. Further studies are warranted to prove this part. Second, the validity of the NNS-FFQ compared with urinary biomarker concentrations only revealed moderate consistency and may have been limited by product availability in the market and the populations’ favourite products containing NNS. Clinical tests can be sensitive in reflecting the dietary intake at a specific point in time or within 24 h of ingestion. DR or poor memory may have been problems in adolescents and children and may have caused validity examination to be less effective.

Overestimates of intake may be an issue when FFQ is applied in a study^(34)^. The risk of overestimates in FFQ generally comes from probably the option of frequency more than once a day and assessed specified portion sizes by populations. To avoid overestimation, ascending closed format of frequency option was arranged in the FFQ. The design of reported specified portion sizes depended on the package or serving size to which each food item was indicated rather than the one classification of the small, medium and large. Furthermore, the photographs of each food were present aside to assist participants to recognise food items and portion size. However, underestimates in the FFQ may be an influential factor. Condiments or spice-containing NNS were not included in the study because the populations usually did not prepare their meals by themselves.

In conclusion, the FFQ was developed to have an online format with thirteen categories and 305 food items for evaluating the consumption of acesulfame potassium, aspartame, sucralose, glycyrrhizin, steviol glycosides and sorbitol. Displaying a photo with text in the measurement was used to increase product recognition because of diverse NNS-containing foods in the Asian market. Sorbitol consumption appeared high compared with other areas worldwide, similar to that of other Asian populations. The NNS consumption pattern in Asia differs from that in Western communities. Therefore, this measurement is crucial for investigations and studies on health concerns in Asia. Significantly moderate validation of this measurement with the urinary biomarker was observed for steviol glycosides and sucralose intake. Therefore, this measurement is applicable and useful for evaluating NNS consumption in epidemiological and clinical studies.

## Supplementary material

For supplementary material accompanying this paper visit https://doi.org/10.1017/S136898002200088X. click here to view supplementary material
