# Supplementary material for: Development and validation of the Chinese version non-nutritive sweetener FFQ with urinary biomarker in children and adolescents
Source: Public Health Nutr. 2022 Apr 13;25(8):2056–63. doi: 10.1017/S136898002200088X (PMC9991608; doi:10.1017/S136898002200088X)
Supplement: Supplementary file 1 [file S136898002200088Xsup.zip › S136898002200088Xsup002.docx]

Table S1. Non-nutritive Sweetener Food Frequency Questionnaire page 1 of 14

Instructions: Participant ID: ______________ Date: ______________

1. Please review each food category indicated below on web page, and recognize what food item was consumed during past month.
2. Indicate how often you consumed the following food item, for example, if you ate potato chips 3 times per week, click 2-3x/week.
3. Indicate how approximate amount you consumed the following food item each time, for example, if you ate potato chips 1 pack each time, click 1 pack.
4. Please make sure you have reviewed all the food categories before you summit the data on the web page.

Chinese version of NNS-FFQ are available from the website: <https://docs.google.com/forms/d/1HPRKMoO4Kc1fGzvz7kiK5bzegVDbDUCQpAcxgA-435E/viewform?chromeless=1&edit_requested=true>

https://docs.google.com/forms/d/e/1FAIpQLSf6X-tr1nzzWMUAVKuEGlnNiZugbkA4BwlRIqY-_jBgJq_CQg/viewform?usp=sf_link

†: 240ml for a bowl; ‡: 1 piece is about 2/3 to 1 of a palm; §; 7-10 g of solid food or 15g of powder food for a spoon

| Table S2. Urinary NNS concentration for children and adolescents in the study | | | | |  |
| --- | --- | --- | --- | --- | --- |
| Urinary NNS | Children / Adolescents | | | | |
|  | NNS concentration (ng/ml)/urine creatinine(mg/ml) | | NNS concentration (ng/ml)/urine creatinine(mg/ml)/body weight | | |
|  | Mean | SD | Mean | SD | |
| Acesulfame potassium | 9021.38 | 27070.82 | 258.46 | 832.48 | |
| Sucralose | 444.17 | 1180.68 | 14.57 | 50.94 | |
| Steviol glycosides | 1720.08 | 4408.64 | 50.28 | 132.94 | |
| SD: standard deviation |  |  |  |  | |

| Table S3. Agreement between two individual FFQ investigations regarding NNS and sorbitol consumption patterns among young adults using Cohen’s Kappa analysis in the pretest study | | | |
| --- | --- | --- | --- |
| NNS type | Number of participants reporting any  consumption through FFQ1 (%) | Number of participants reporting any  consumption through FFQ2 (%) | Cohen’s κ  （FFQ 1 vs FFQ2） |
| Acesulfame Potassium | 54% | 46% | 0.69 |
| Aspartame | 62% | 69% | 0.49 |
| Sucralose | 62% | 58% | 0.76 |
| Glycyrrhizin | 31% | 35% | 0.35 |
| Steviol glycosides | 31% | 23% | 0.61 |
| Sorbitol | 69% | 69% | 0.64 |
| Added sugar | 58% | 58% | 0.37 |
| NNS: non-nutritive sweetener; FFQ: food frequency questionnaire | | | |

| Table S4. Descriptive statistics and Spearman’s correlation for two individual FFQs among young adults in the pretest study | | | | | | | | |
| --- | --- | --- | --- | --- | --- | --- | --- | --- |
| NNS type | NNS-FFQ1  (mg, mean) | SD | NNS-FFQ2  (mg, mean) | SD | Spearman’s Correlation  (ρ) | *p* value | Mean Difference  (mg, mean) | SD |
| Acesulfame Potassium | 1.48 | 3.31 | 0.62 | 1.37 | 0.52 | 0.01 | 0.86 | 3.49 |
| Aspartame | 1.99 | 4.70 | 0.61 | 1.12 | 0.49 | 0.01 | 1.38 | 4.83 |
| Sucralose | 2.01 | 3.93 | 0.67 | 0.07 | 0.70 | <0.001 | 1.34 | 3.95 |
| Glycyrrihizin | 0.05 | 0.08 | 0.04 | 0.07 | 0.36 | 0.07 | 0.02 | 0.08 |
| Steviol | 0.16 | 0.35 | 0.08 | 0.26 | 0.50 | 0.01 | 0.08 | 0.43 |
| Sorbitol | 439.95 | 614.54 | 412.44 | 660.86 | 0.67 | <0.001 | 27.50 | 474.76 |
| Added sugar | 5.35 | 12.08 | 3.73 | 6.84 | 0.34 | 0.09 | 1.62 | 14.04 |
| NNS: non-nutritive sweetener; FFQ: food frequency questionnaire; SD: standard deviation  The mean and SD of sorbitol consumption in children and adolescents from FFQ1 report were 110.30 mg and 176.78 mg, respectively. | | | | | | | | |

| Table S5. The number of food items containing NNSs and sorbitol reported in the questionnaire | | | | | | |
| --- | --- | --- | --- | --- | --- | --- |
| NNS  Food item | Acesulfame potassium | Aspartame | Sucralose | Glycyrrhizin | Steviol glycosides | Sorbitol |
| Potato chips, corn flakes, cookies, biscuits, and desserts |  | 18 | 25 | 11 | 6 | 17 |
| Beverages | 11 | 5 | 11 |  | 4 | 1 |
| Seaweed |  |  |  | 4 |  |  |
| Chewing gum, candy, and jelly | 7 | 3 | 3 |  | 2 | 33 |
| Dehydrated minced seafoods | 2 | 1 | 2 | 3 |  | 37 |
| Frozen foods | 6 | 1 | 7 | 13 | 1 | 36 |
| Preserved fruits | 2 | 2 | 10 | 5 | 2 | 1 |
| Jerky, dried tofu, and eggs |  |  | 2 | 2 |  | 16 |
| Nutritional supplements |  |  | 6 |  |  | 2 |
| Seasoned seeds and nuts | 5 | 1 | 2 | 1 |  |  |
| Instant noodles |  |  |  |  |  | 7 |
| Sugar substitutes |  | 1 | 3 |  | 1 |  |
| Energy drinks | 1 |  | 3 |  |  |  |
| Total | 34 | 32 | 74 | 39 | 16 | 150 |

(c) (d)

(a) (b)

Figure S1. Indicated NNS concentration at baseline and 2, 5, 10, and 24 hours after either high- or low-dose treatment. Concentrations are those for (a) acesulfame potassium, (b) sucralose, and (c) steviol glycosides.
